# Study on mechanism of action of β-elemene in inhibiting cisplatin resistance in lung cancer through LncRNA LINC00511

**DOI:** 10.3389/fphar.2025.1586657

**Published:** 2025-12-17

**Authors:** Xiaoli Deng, Chunjie Hu, Lin Li, Guixian Yang, Chikun Li, Wanxun Zhang, Guangyu Cheng

**Affiliations:** 1 School of Pharmacy, Changchun University of Chinese Medicine, Changchun, China; 2 Anorectal Center, The Affiliated Hospital to Changchun University of Chinese Medicine, Changchun, China; 3 Healthcare Department, The Affiliated Hospital to Changchun University of Chinese Medicine, Changchun, China; 4 Teaching Department, The Third Affiliated Clinical Hospital of Changchun University of Chinese Medicine, Changchun, China; 5 The Research Center of The Affiliated Hospital to Changchun University of Chinese Medicine, Changchun, China

**Keywords:** lung cancer, β-elemene, cisplatin resistance, LINC00511, PI3K/AKT/mTOR pathway

## Abstract

**Background:**

Lung cancer is a leading cause of cancer-related deaths, with cisplatin being a cornerstone of treatment. However, resistance to cisplatin presents a significant challenge. β-elemene, a natural compound, has demonstrated potential to reverse cisplatin resistance. LncRNA LINC00511 has been implicated in cisplatin resistance through its role in activating the PI3K/AKT/mTOR pathway, which supports tumor survival and proliferation.

**Purpose:**

This study aims to investigate the mechanism by which β-elemene overcomes cisplatin resistance in lung cancer by regulating LINC00511.

**Methods:**

Human lung adenocarcinoma cells (A549 and A549/DDP) were treated with β-elemene and cisplatin. Cell proliferation and apoptosis were assessed using CCK-8, EdU staining, and flow cytometry. LINC00511 expression was measured by qRT-PCR, and protein levels of PI3K, AKT, and mTOR were evaluated via Western blot. A xenograft model was used to confirm *in vivo* effects.

**Results:**

β-elemene significantly enhanced cisplatin-induced apoptosis in A549/DDP cells, reduced LINC00511 expression, and inhibited the PI3K/AKT/mTOR pathway. LINC00511 knockdown further potentiated these effects, both *in vitro* and *in vivo*. Xenograft models confirmed the enhanced anti-tumor effects of the combination treatment.

**Conclusion:**

β-elemene overcomes cisplatin resistance in lung cancer by downregulating LINC00511 and inhibiting the PI3K/AKT/mTOR pathway. These findings propose a promising therapeutic strategy for treating cisplatin-resistant lung cancer.

## Graphical Abstract

**Figure:**
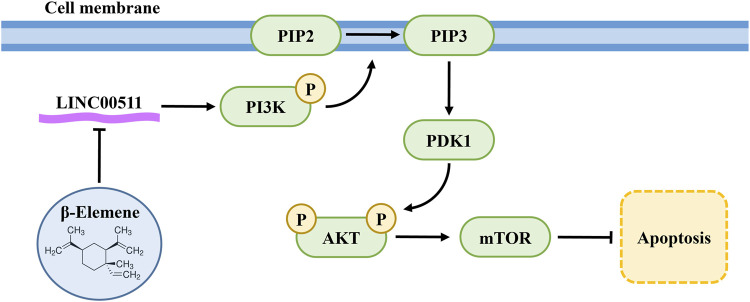

## 1 Introduction

Lung cancer remains one of the most prevalent and lethal malignancies globally, accounting for over 2.2 million new cases annually (Leiter et al., 2023). This malignancy is classified into two main types: non-small cell lung cancer (NSCLC), which represents around 85% of cases, and small cell lung cancer (SCLC), which accounts for the remaining 15% (Rudin et al., 2021; Papavassiliou and Papavassiliou, 2022). The primary causes of lung cancer include exposure to carcinogens like tobacco smoke, genetic predisposition, and environmental factors such as air pollution and asbestos (Malhotra et al., 2016; Megyesfalvi et al., 2023; Wang Q. et al., 2023). Despite advancements in screening and diagnostic technologies, early detection remains challenging, and the disease is often diagnosed at an advanced stage (Saab et al., 2022; Chang et al., 2024).

Advances in understanding the genetic landscape of lung cancer have facilitated the development of targeted therapies, revolutionizing its treatment (Hirsch et al., 2017). Mutations in key oncogenes such as the epidermal growth factor receptor (EGFR) and rearrangements in the anaplastic lymphoma kinase (ALK) have led to the development of precision therapies, significantly improving outcomes in patients harboring these mutations (da Cunha Santos et al., 2011; Rodriguez Abreu et al., 2024). Targeted therapies, such as tyrosine kinase inhibitors (TKIs) directed against EGFR or ALK, have shown remarkable efficacy in selected patients (Tan and Tan, 2022). However, acquired resistance to these treatments remains a significant challenge, necessitating further research into overcoming therapeutic resistance.

Platinum-based chemotherapeutic agents, particularly cisplatin and carboplatin, have been the cornerstone of lung cancer treatment for decades (Dasari and Tchounwou, 2014). These drugs exert their cytotoxic effects by forming DNA crosslinks, leading to apoptosis in rapidly dividing cells (Herzog et al., 2021). Cisplatin, one of the earliest platinum-based drugs introduced, is used in combination with other chemotherapeutic agents like paclitaxel or gemcitabine for the treatment of NSCLC (Rose et al., 2014). Similarly, carboplatin, a cisplatin analog, has been widely adopted due to its comparable efficacy and more favorable side effect profile, particularly in patients who cannot tolerate the nephrotoxicity associated with cisplatin (Chaft et al., 2021). Despite the efficacy of platinum compounds, their use is often limited by the development of drug resistance (Zhong et al., 2020). Tumor cells can acquire resistance through several mechanisms, including enhanced DNA repair, drug efflux, and detoxification systems (Robbrecht et al., 2024). This has led to ongoing research into novel platinum derivatives and combination therapies aimed at improving the therapeutic index of these drugs (Zheng et al., 2024).

β-elemene, a sesquiterpene compound extracted from the traditional Chinese medicinal plant *Curcuma zedoaria*, has gained significant attention for its anticancer properties (Luo et al., 2019). It exhibits broad-spectrum antitumor activity, affecting various cancer types such as lung cancer, breast cancer, and cervical cancer (Li et al., 2020; Feng et al., 2024; Bao et al., 2025). The mechanisms underlying its antitumor effects are multifaceted, involving the induction of apoptosis, inhibition of proliferation, suppression of metastasis, and modulation of the immune response. β-elemene has also been shown to induce autophagy in cancer cells, contributing to its therapeutic effects (Wu et al., 2024). The potential of β-elemene becomes particularly interesting when considered in combination with platinum-based chemotherapy. β-elemene has been found to sensitize cancer cells to cisplatin, thereby enhancing the efficacy of the treatment (Qian et al., 2024). This synergy is primarily attributed to β-elemene’s ability to modulate key molecular pathways involved in drug resistance, such as the PI3K/AKT/mTOR axis, which plays a role in cisplatin resistance in NSCLC cells (Cheng et al., 2022).

Long noncoding RNAs (lncRNAs) are a class of RNA molecules longer than 200 nucleotides that do not encode proteins. They have emerged as critical regulators of gene expression, involved in diverse cellular processes such as chromatin remodeling, transcriptional regulation, and post-transcriptional modification (Kazimierczyk and Wrzesinski, 2021). Unlike protein-coding RNAs, lncRNAs function primarily by interacting with DNA, RNA, and proteins, forming complexes that influence chromatin structure and gene expression. LncRNAs can act as molecular scaffolds, decoys, or guides to regulate gene expression in both cis and trans modes (Chen et al., 2024). Recent research has highlighted the role of lncRNAs in cancer biology, where they function as oncogenes or tumor suppressors (Santos et al., 2020). In lung cancer, lncRNAs have been shown to play pivotal roles in tumor initiation, progression, and metastasis (Wang M. et al., 2023). LncRNAs like HOTAIR, MALAT1, and LINC01234 have been found to be aberrantly expressed in lung cancer tissues, where they promote cancer cell proliferation, metastasis, and resistance to apoptosis (Maqbool et al., 2024).

In this study, we identified a significant association between cisplatin resistance and elevated LINC00511 expression, coupled with activation of the PI3K/AKT/mTOR pathway in lung cancer cells. We demonstrated that β-elemene, when used in combination with cisplatin, effectively reduces cell proliferation and promotes apoptosis in cisplatin-resistant A549/DDP cells. This combination therapy further enhances the cytotoxic effects of cisplatin by downregulating LINC00511 expression and Inhibiting the PI3K/AKT/mTOR signaling pathway. Knockdown of LINC00511 intensifies the pro-apoptotic effects, indicating its key role in mediating cisplatin resistance. Our findings underscore the therapeutic potential of β-elemene in overcoming cisplatin resistance in lung cancer through the regulation of LINC00511 and the PI3K/AKT/mTOR pathway, offering new insights into treatment strategies for chemoresistant lung cancer.

## 2 Methods

### 2.1 Cell culture

The human lung adenocarcinoma A549 cell line and its cisplatin-resistant variant, A549/DDP, were purchased from Pricella (Wuhan, China). Both cell lines were initially thawed in a 37 °C water bath and transferred into cell culture flasks for further expansion. A549 cells were cultured in Ham’s F-12K medium supplemented with 10% fetal bovine serum (FBS) and 1% penicillin-streptomycin (P/S). A549/DDP cells were cultured in Ham’s F-12K medium supplemented with 10% FBS, 1–2 μg/mL cisplatin (DDP), and 1% P/S. All cells were maintained in a humidified incubator at 37 °C with 5% CO_2_.

### 2.2 Reagents and antibodies

Cisplatin (catalog #C489606) was purchased from Aladdin (Shanghai, China), and β-elemene (catalog #63965) was obtained from MilliporeSigma (Darmstadt, Germany). LY294002 (catalog #HY-10108), a PI3K inhibitor, was sourced from MedChemExpress (Monmouth Junction, NJ, United States). Cell viability was assessed using the Cell Counting Kit-8 (CCK-8, catalog #BS350B) from Biosharp (Hefei, China). The Annexin V-FITC Apoptosis Detection Kit (catalog #AP101) was obtained from MultiSciences (Hangzhou, China), and the EdU Cell Proliferation Kit (catalog #C0071S) was purchased from Beyotime (Shanghai, China). Apoptotic cells were detected using the Fluorescein (FITC) TUNEL Cell Apoptosis Detection Kit (catalog #G1501) from Servicebio (Wuhan, China). For Western blot analysis, the following antibodies were used: BAD antibody (1:500), Bcl-2 antibody (1:2000), Cleaved-Caspase 3 antibody (1:500), and Phospho-PI3K p85 antibody (1:500), all from Lingjiesi Biotech (Wuhan, China). Additional antibodies included AKT monoclonal antibody (1:5000), Phospho-AKT (Ser473) antibody (1:1000), PI3K p85 Alpha antibody (1:5000), Phospho-mTOR (Ser2448) monoclonal antibody (1:2000), and mTOR monoclonal antibody (1:5000), all from Proteintech (Wuhan, China). The Ki67 antibody (1:300) was obtained from Servicebio (Wuhan, China). Lipofectamine™ 2000, used for transfections, was purchased from Thermo Fisher Scientific (Waltham, MA, United States).

### 2.3 Cell viability assay

Cell viability was assessed using the CCK-8 assay (#BS350B, Biosharp, Hefei, China). A549 cells were divided into groups: control, DDP (4 μM, 8 μM), β-elemene (40 μg/mL) + DDP (4 μM, 8 μM), and β-elemene (80 μg/mL) + DDP (4 μM, 8 μM). A549/DDP cells were divided similarly: control, DDP (12 μM, 24 μM), β-elemene (40 μg/mL) + DDP (12 μM, 24 μM), and β-elemene (80 μg/mL) + DDP (12 μM, 24 μM). Cells were seeded in 96-well plates at a density of 1 × 10^4^ cells per well (n = 3 per group) and treated with the corresponding β-elemene and DDP concentrations after overnight incubation. Following 24 h treatment at 37 °C with 5% CO_2_, 10 μL of CCK-8 solution was added to each well and incubated for 1 h. Absorbance at 450 nm was measured using a microplate reader (DeTieLab, Nanjing, China) to determine cell viability.

### 2.4 5-Ethynyl-2′-deoxyuridine (EdU) incorporation assay

Cell proliferation was assessed using an EdU assay kit (#C0071, Beyotime, Shanghai, China). Cells (1 × 10^5^ cells/well) were seeded into 24-well plates and treated as per experimental groups. EdU (10 mM) was diluted 1:500 in culture medium to prepare a 2× EdU working solution (20 μM), which was prewarmed and added to the cells, yielding a final concentration of 1×. After a 2-h incubation at 37 °C, cells were fixed with 4% paraformaldehyde for 15 min, washed with PBS, and permeabilized with 0.3% Triton X-100. The Click reaction solution was then added, and the cells were incubated in the dark for 30 min. After washing, nuclei were stained with Hoechst 33342 for 10 min. Cells were observed under a fluorescence microscope, with Hoechst 33342 exhibiting blue fluorescence (excitation 346 nm, emission 460 nm).

### 2.5 Flow cytometry

Apoptosis was evaluated by flow cytometry following a previously described protocol with minor modifications (Gupta et al., 2023). Briefly, Cells (1 × 10^6^), including those collected from the culture supernatant, were harvested and washed with pre-cooled PBS via centrifugation. The cells were resuspended in 500 μL of 1× Binding Buffer, prepared by diluting 5× Binding Buffer with distilled water. Each sample was then treated with 5 μL of Annexin V-FITC and 10 μL of propidium iodide (PI). After gentle vortexing, samples were incubated in the dark at room temperature for 5 min. Flow cytometric analysis was performed, detecting Annexin V-FITC via the FITC channel (Ex = 488 nm; Em = 530 nm) and PI via the PI channel (Ex = 535 nm; Em = 615 nm).

### 2.6 Western blot analysis

A549/DDP cells were seeded in 6-well plates at a density of 1 × 10^6^ cells per well and treated for 48 h. After treatment, cells were collected by centrifugation and lysed on ice using RIPA buffer supplemented with 1 mM PMSF for 30 min. The lysates were then centrifuged at 12,000 rpm for 10 min at 4 °C, and the supernatant protein concentrations were determined using the BCA assay. Proteins were denatured, separated by 10%–15% SDS-PAGE, and transferred onto PVDF membranes. The membranes were blocked for 2 h and subsequently incubated with primary antibodies overnight at 4 °C. After washing, membranes were incubated with appropriate secondary antibodies for 1 h at room temperature. Protein bands were detected using enhanced chemiluminescence (ECL) reagents. The Western blot procedure was performed in accordance with established methodologies (Akhtar et al., 2024).

### 2.7 Quantitative reverse transcriptase-polymerase chain reaction (RT-qPCR)

Total RNA was extracted from A549/DDP cells using the Ultrapure RNA Kit (#CW0597S, Cwbio, Taizhou, China) according to the manufacturer’s instructions. The extracted RNA was reverse-transcribed into complementary DNA (cDNA) for subsequent qPCR amplification. Gene-specific primers were designed, and their sequences are provided in Table 1. GAPDH was used as an internal reference gene to normalize the expression of target genes. Quantitative RT-PCR was performed as previously described (El-Aarag et al., 2014), and relative gene expression levels between the control and experimental groups were calculated using the 2^−ΔΔCT^ method.

**TABLE 1:** Primer sequence listing.

Primer	Sequence (5′-3′)
LINC00511-F	TTT​CCC​AGC​ACA​GCT​CAA​TC
LINC00511-R	TCCCTTCTCCCTCGGTCA
MBNL1-AS1-F	TCC​CTA​CAC​TCA​AGG​ATA​ACG
MBNL1-AS1-R	TTG​GAT​TGC​TTC​CCA​CAT​A
PSMA3-AS1-F	CGT​TTC​CTC​CAG​GAC​AGC​AC
PSMA3-AS1-R	TCG​CAG​ATC​CAG​GTT​TCT​CAA
TRAM2-AS1-F	GCT​GAG​ACC​TCC​TGC​GAA​CA
TRAM2-AS1-R	GAT​GTC​ATC​TGA​AGG​CTT​AAC​TGG
LINC00630-F	CGCTCTGGCTGTTTCGTG
LINC00630-R	CAA​TCT​GGC​AAA​GAG​GGA​CT
GAPDH-F	AAT​CCC​ATC​ACC​ATC​TTC​CA
GAPDH-R	AAA​TGA​GCC​CCA​GCC​TTC​T

### 2.8 Immunohistochemical (IHC) analysis

Immunohistochemical staining for Ki-67 was carried out to evaluate cell proliferation in tumor tissue sections, in accordance with established methodology (El-Saied et al., 2019). Briefly, tumor samples were fixed in 10% formaldehyde, embedded in paraffin, and sliced into 5 μm sections. After deparaffinization and rehydration, antigen retrieval was performed using citrate buffer. Endogenous peroxidase activity was quenched by treatment with 3% hydrogen peroxide. The sections were then incubated overnight at 4 °C with an anti-Ki-67 primary antibody (Servicebio, Wuhan, China). This was followed by incubation with a secondary antibody and development with DAB substrate. Finally, the sections were counterstained with hematoxylin, mounted, and examined under a microscope.

### 2.9 Tumor xenograft model *in vivo*

All animal experiments were approved and performed in accordance with the guidelines of the Animal Care and Welfare Committee of Changchun University of Chinese Medicine (approval no. 2023409). The experiments were also conducted in compliance with the National Institute of Health Guidelines for the Care and Use of Laboratory Animals, the European Community Council Directive of November 2010 for Care, and Use of Laboratory Animals (Directive 2010/63/EU) guidelines. All procedures were performed to minimize animal use and alleviate suffering.

4- to 6-week-old male BALB/c nude mice (purchased from Hangzhou Ziyuan Experimental Animal Technology Co., Ltd.) were acclimated for 7 days. A total of 28 mice were injected with 1 × 10^7^ A549/DDP cells resuspended in 100 μL PBS into the right axilla. Once tumors reached approximately 30 mm^3^, mice were randomly assigned to 4 groups: Control, DDP (cisplatin, 5 mg/kg), β-elemene (75 mg/kg) (Zheng et al., 2018), and DDP+β-elemene, with 7 mice per group. Treatments were administered daily via intraperitoneal injection for 21 days. Tumor size was measured every 2 days with calipers, and body weight was recorded. At the end of the treatment, the mice were euthanized, and tumors were excised, weighed, and photographed. Half of each tumor was fixed in 4% paraformaldehyde for histological analysis, while the other half was frozen at −80 °C for further use.

### 2.10 Statistical analysis

All experiments were independently repeated 3 times to ensure the reliability and reproducibility of the results. Data are presented as the mean ± standard deviation (SD). Statistical analysis was conducted using GraphPad Prism 8 software (GraphPad Software, USA). One-way analysis of variance (One-Way ANOVA) was used to compare means among multiple groups, and the significance of differences was determined by Tukey’s *post hoc* test. A p-value of less than 0.05 was considered statistically significant. ImageJ software (NIH, United States) was used for the quantitative analysis of Western blot and immunofluorescence staining results.

## 3 Results

### 3.1 β-Elemene enhances cisplatin sensitivity and induces apoptosis in A549 and A549/DDP cells

**FIGURE 1:**
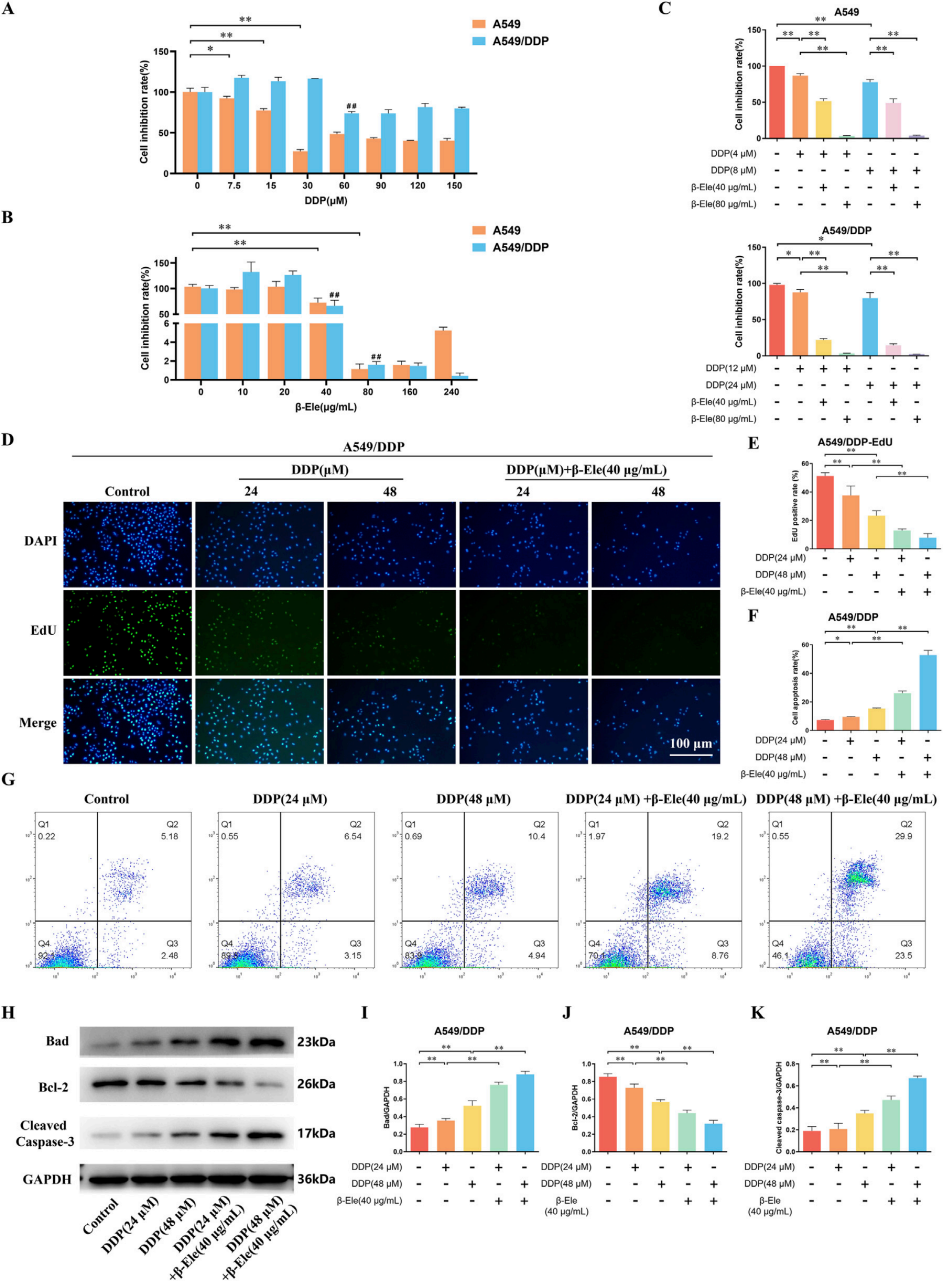
β-Elemene Enhances Cisplatin Sensitivity and Induces Apoptosis in A549 and A549/DDP Cells. **(A)** The IC50 of cisplatin on A549 and A549/DDP cells as determined by CCK-8 assay; **(B)** The IC50 of β-elemene on A549 and A549/DDP cells; **(C)** The combined inhibitory effects of cisplatin and β-elemene on A549 and A549/DDP cell proliferation; **(D,E)** EdU staining results of A549/DDP Cells; **(F,G)** Flow cytometry analysis of A549/DDP Cells; **(H–K)** Western blot analysis of apoptosis-related proteins in A549/DDP cells. Data are presented as mean ± SD from three independent experiments (biological replicates). ^*^ *P* < 0.05, ^**^ *P* < 0.01, ^##^ *P* < 0.01.

We assessed the inhibitory effects of cisplatin and β-elemene on the proliferation of A549 cells and their cisplatin-resistant counterpart, A549/DDP. The results demonstrated that both agents significantly suppressed cell proliferation in a dose-dependent manner. Specifically, the half-maximal inhibitory concentration (IC50) of cisplatin was 16 μM for A549 cells and 48 μM for A549/DDP cells (*P* < 0.01) (Figure 1A), indicating a marked resistance in the A549/DDP line. In contrast, the IC50 of β-elemene was 80 μg/mL for both A549 and A549/DDP cells (*P* < 0.01) (Figure 1B), suggesting that β-elemene retained comparable inhibitory efficacy in both cell types. Subsequent experiments were conducted using concentrations corresponding to 1/4 and 1/2 of the respective IC50 values. When cisplatin and β-elemene were administered in combination, a significantly enhanced inhibitory effect on cell proliferation was observed in both cell lines, with a particularly notable suppression in the A549/DDP cells (*P* < 0.01) (Figure 1C).

Further investigation using EdU staining demonstrated that the proliferation of A549/DDP cells was notably inhibited following treatment with cisplatin, and this inhibitory effect was further enhanced in the group receiving the combination of cisplatin and β-elemene (*P* < 0.01) (Figures 1D,E). Consistently, flow cytometry analysis revealed a significant increase in apoptosis in A549/DDP cells treated with cisplatin alone, with an even more pronounced apoptotic response observed in the combination treatment group (*P* < 0.01) (Figures 1F,G).

Western blot analysis of apoptosis-related proteins showed that, compared to the control group, cisplatin treatment led to a marked upregulation of Bad and Cleaved Caspase-3 expression levels, along with a downregulation of Bcl-2 in A549/DDP cells. Furthermore, compared to cisplatin treatment alone, the combination of β-elemene and cisplatin significantly increased the expression of Bad and Cleaved Caspase-3, while further reducing Bcl-2 expression in A549/DDP cells (*P* < 0.01) (Figures 1H–K). These results suggest that β-elemene enhances the cisplatin-induced apoptotic response and overcomes cisplatin resistance in A549/DDP cells.

### 3.2 β-Elemene modulates LncRNA expression profiles to overcome cisplatin resistance in A549/DDP cells

**FIGURE 2:**
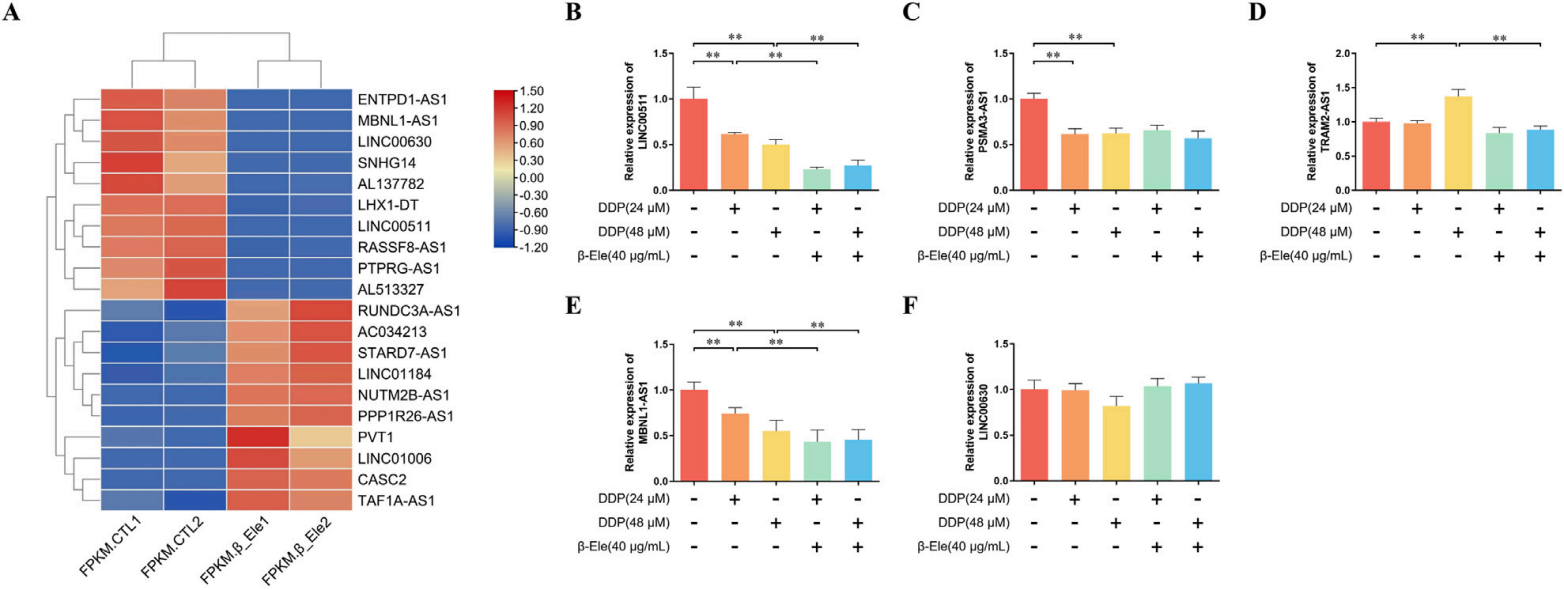
β-Elemene Modulates LncRNA Expression Profiles to Overcome Cisplatin Resistance in A549/DDP Cells. **(A)** LncRNA sequencing analysis showing differentially expressed LncRNAs between control and β-elemene-treated A549/DDP cells. Each condition represents two independent biological replicates; **(B–F)** qRT-PCR validation of the expression levels of LINC00511, PSMA3-AS1, TRAM2-AS1, LINC00630, and MBNL1-AS1 in A549/DDP cells. Data are presented as mean ± SD of three independent biological replicates per group. ^**^ *P* < 0.01.

LncRNA sequencing analysis revealed significant differences in the expression levels of several LncRNAs between the control group and β-elemene-treated A549/DDP cells, suggesting their potential involvement in β-elemene-mediated regulation of cisplatin resistance. Notably, among the differentially expressed lncRNAs, LINC00511, PSMA3-AS1, TRAM2-AS1, LINC00630, and MBNL1-AS1 exhibited distinct expression patterns, with LINC00511 showing the most significant change (Figure 2A).

To validate these findings, the expression levels of LINC00511, PSMA3-AS1, TRAM2-AS1, LINC00630, and MBNL1-AS1 were further quantified using qRT-PCR. The results confirmed that β-elemene significantly downregulated the expression of LINC00511 in A549/DDP cells (*P* < 0.01) (Figures 2B–F). These data suggest that LINC00511 may play a key role in the mechanism by which β-elemene modulates cisplatin resistance in lung cancer cells.

The discrepancy in LINC00630 expression between sequencing and qRT-PCR analyses may be attributed to several factors inherent in high-throughput methodologies, such as potential false positives arising from multiple hypothesis testing, challenges in quantifying low-abundance transcripts, or technical variations in sample preparation and analysis pipelines. It is also plausible that LINC00630 may have subtly changed at a specific splice variant level not effectively captured by our qRT-PCR primer design.

### 3.3 β-Elemene potentiates the antitumor effects of cisplatin in A549/DDP xenograft models by inhibiting tumor growth and promoting apoptosis

**FIGURE 3:**
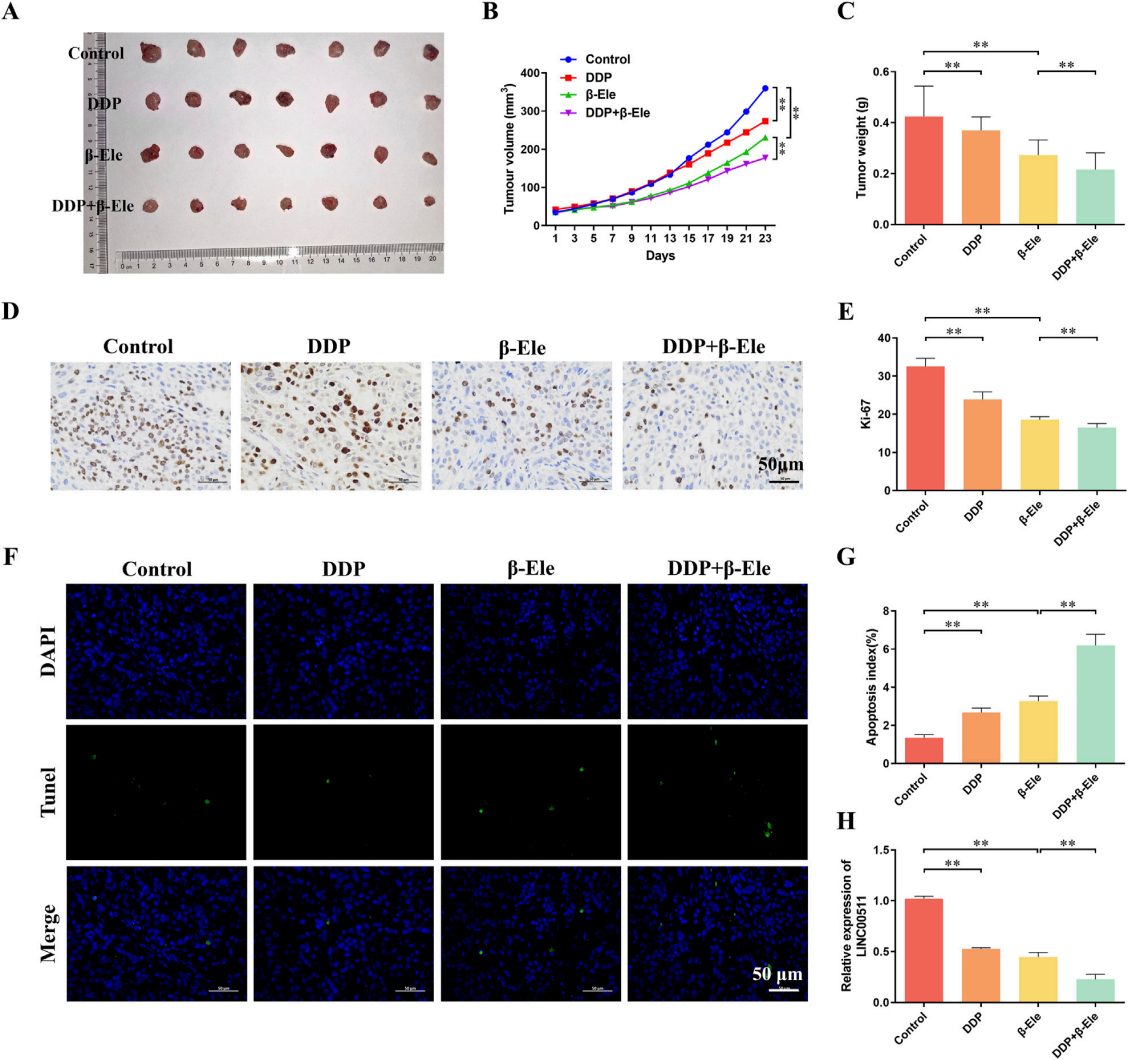
β-Elemene Potentiates the Antitumor Effects of Cisplatin in A549/DDP Xenograft Models by Inhibiting Tumor Growth and Promoting Apoptosis. **(A–C)** Tumor mass analysis in A549/DDP xenograft models treated with cisplatin, β-elemene, or the combination of both; **(D,E)** Immunohistochemical staining for Ki-67 in xenograft tumor tissues; **(F,G)** TUNEL staining for apoptosis detection in xenograft tumors; **(H)** qRT-PCR analysis of LINC00511 expression in tumor tissues. All experiments were independently repeated seven times (biological replicates). Data are presented as mean ± SD. ^**^ *P* < 0.01.

The antitumor efficacy of β-elemene in combination with cisplatin was evaluated *in vivo* using a xenograft model of A549/DDP cells in nude mice. Tumor mass analysis revealed that the cisplatin-treated group exhibited a tumor weight of 0.37 ± 0.05 g, which was lower than that of the control group (0.42 ± 0.12 g). The β-elemene-treated group showed a more significant reduction in tumor mass (0.27 ± 0.06 g) compared to the control. Notably, the combination of β-elemene and cisplatin resulted in the most pronounced tumor suppression, with a tumor weight of 0.22 ± 0.07 g (*P* < 0.01) (Figures 3A–C). Immunohistochemical staining for Ki-67, a marker of cell proliferation, demonstrated a marked reduction in the number of Ki-67-positive cells in all treatment groups compared to the control. The lowest number of Ki-67-positive cells was observed in the combination treatment group, indicating enhanced suppression of tumor cell proliferation (*P* < 0.01) (Figures 3D,E). Apoptosis was assessed using TUNEL staining, which revealed significantly increased apoptotic cell percentages in all treatment groups compared to the control. The highest apoptosis rate was observed in the combination treatment group, while the cisplatin-only group exhibited the lowest rate of apoptosis among the treated groups (*P* < 0.01) (Figures 3F,G). Further analysis by qRT-PCR showed that LINC00511 expression was significantly reduced in all treatment groups, with the most pronounced downregulation observed in the combination treatment group compared to the cisplatin-only group (*P* < 0.01) (Figure 3H). These results suggest that β-elemene enhances the antitumor effects of cisplatin by reducing tumor growth, promoting apoptosis, and downregulating LINC00511 expression in A549/DDP xenografts.

### 3.4 LINC00511 modulates the apoptosis and proliferation of A549/DDP cells

To further elucidate the role of LINC00511 in β-elemene-mediated enhancement of cisplatin sensitivity, A549/DDP cells were subjected to LINC00511 overexpression or knockdown. The cells were divided into eight groups: control, β-elemene, β-elemene + LINC00511 overexpression, β-elemene + LINC00511 knockdown, cisplatin, cisplatin + β-elemene, cisplatin + β-elemene + LINC00511 overexpression, and cisplatin + β-elemene + LINC00511 knockdown.

**FIGURE 4:**
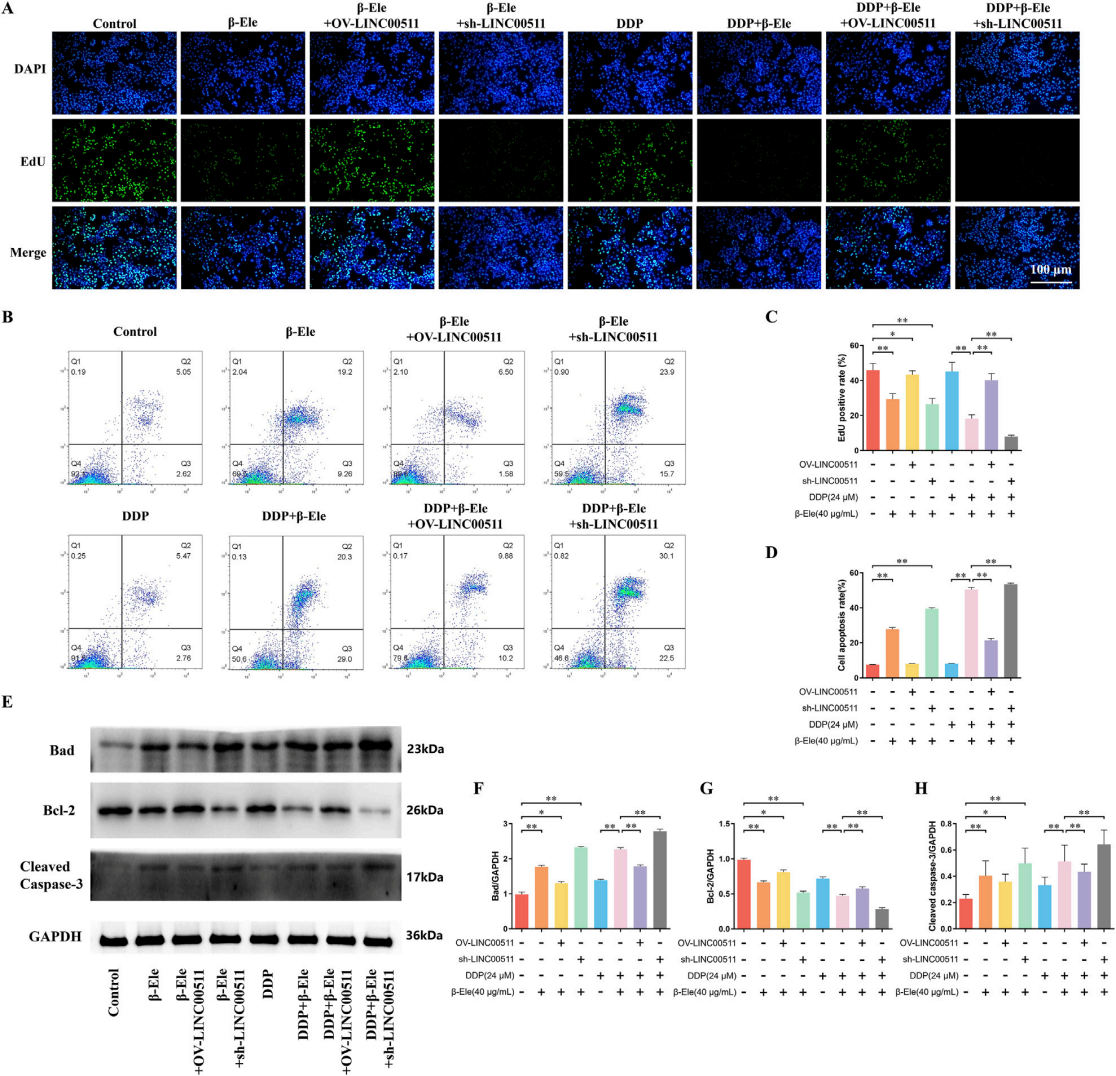
LINC00511 Modulates the Apoptosis and Proliferation of A549/DDP Cells. **(A,C)** EdU staining analysis of cell proliferation in A549/DDP cells across different treatment groups; **(B,D)** Flow cytometry analysis of apoptosis in A549/DDP cells; **(E–H)** Western blot analysis of apoptosis-related proteins. Data are presented as mean ± SD from three independent experiments (biological replicates). ^*^ *P* < 0.05, ^**^ *P* < 0.01.

EdU staining results showed that β-elemene treatment significantly reduced cell proliferation compared to the control group. In contrast, LINC00511 overexpression partially rescued the β-elemene-induced suppression of proliferation, whereas LINC00511 knockdown in combination with β-elemene led to a further reduction in proliferation. The cisplatin group exhibited a modest decrease in proliferation, but the combination of β-elemene with cisplatin led to a more pronounced inhibitory effect. Notably, the greatest suppression of cell proliferation was observed in the cisplatin + β-elemene + LINC00511 knockdown group, highlighting the synergistic effect of LINC00511 knockdown (*P* < 0.01) (Figures 4A,C). Flow cytometry analysis of apoptosis revealed similar trends. β-elemene treatment significantly increased apoptosis, while LINC00511 overexpression mitigated this effect. In contrast, LINC00511 knockdown in combination with β-elemene further enhanced apoptosis. Cisplatin alone induced only a modest increase in apoptosis, but when combined with β-elemene, apoptosis was significantly elevated. The highest apoptotic rate was seen in the cisplatin + β-elemene + LINC00511 knockdown group, indicating that LINC00511 knockdown enhances the pro-apoptotic effects of the combination treatment (*P* < 0.01) (Figures 4B,D). Western blot analysis of apoptosis-related proteins showed that Bad and Cleaved Caspase-3 expression was significantly increased in the β-elemene-treated group compared to the control. This effect was also observed in the LINC00511 overexpression group, though to a lesser extent. In contrast, LINC00511 knockdown further elevated Bad and Cleaved Caspase-3 expression in combination with β-elemene or cisplatin. The highest levels of Bad and Cleaved Caspase-3 were detected in the cisplatin + β-elemene + LINC00511 knockdown group, while Bcl-2 expression followed an inverse pattern, with the lowest expression observed in the same group (*P* < 0.01) (Figures 4E–H). These results suggest that LINC00511 plays a critical role in modulating both cell proliferation and apoptosis in response to β-elemene and cisplatin, with knockdown of LINC00511 significantly enhancing the therapeutic effects of the combination treatment.

### 3.5 β-Elemene inhibits cisplatin resistance in A549/DDP cells via downregulation of the PI3K/AKT/mTOR pathway mediated by LINC00511

**FIGURE 5:**
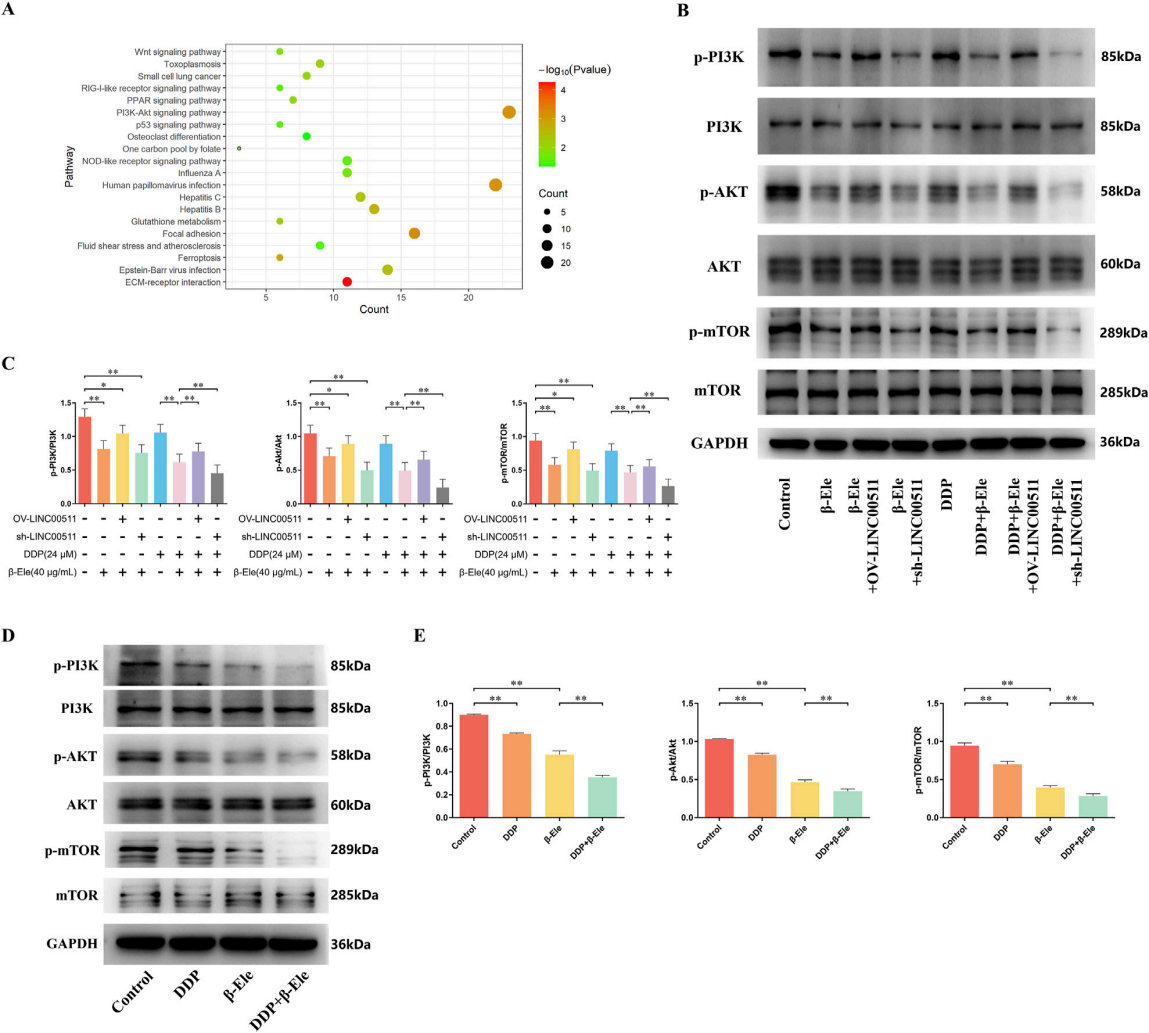
β-Elemene Inhibits Cisplatin Resistance in A549/DDP Cells via Downregulation of the PI3K/AKT/mTOR Pathway Mediated by LINC00511. **(A)** Transcriptomic analysis showing that β-elemene significantly inhibits the activation of the PI3K/AKT/mTOR signaling pathway in A549/DDP cells; **(B,C)** Western blot analysis of phosphorylated and total PI3K, AKT, and mTOR in A549/DDP cells; **(D,E)** Western blot analysis of phosphorylated PI3K, AKT, and mTOR in A549/DDP xenograft tumor tissues. Data are presented as mean ± SD from three independent experiments (biological replicates). ^*^ *P* < 0.05, ^**^ *P* < 0.01.

To investigate the mechanism by which β-elemene reverses cisplatin resistance in A549/DDP cells, transcriptomic analysis was conducted. The results demonstrated that β-elemene significantly inhibited the activation of the PI3K/AKT/mTOR signaling pathway in A549/DDP cells (Figure 5A). In the cell groups established in the previous experiment (Result 3.4), we compared the ratios of phosphorylated to total PI3K, AKT, and mTOR levels. The results indicated a consistent trend across all three proteins. Compared to the control group, the phosphorylation ratios were significantly reduced in the β-elemene treatment group and further decreased in the β-elemene + LINC00511 knockdown group. These ratios were also slightly decreased in the LINC00511 overexpression group. In the cisplatin treatment group, the phosphorylation ratios were similarly reduced, and the combination of cisplatin and β-elemene led to an even more pronounced reduction. Importantly, compared to the cisplatin + β-elemene group, the phosphorylation ratios increased in the cisplatin + β-elemene + LINC00511 overexpression group but were significantly lower in the LINC00511 knockdown group, where the ratios were the lowest among all groups (*P* < 0.01) (Figures 5B,C).

In the A549/DDP xenograft tumor model, Western blot analysis revealed that while the total protein levels of PI3K, AKT, and mTOR remained unchanged across all treatment groups, the levels of their phosphorylated forms (p-PI3K, p-AKT, p-mTOR) were notably altered. Both the cisplatin and β-elemene treatment groups showed a significant reduction in p-PI3K, p-AKT, and p-mTOR levels compared to the control group. Moreover, the combination of cisplatin and β-elemene resulted in a further reduction in the phosphorylation of these proteins compared to cisplatin alone (*P* < 0.01) (Figures 5D,E).

These data indicate that β-elemene suppresses the PI3K/AKT/mTOR pathway activation by reducing the phosphorylation of key proteins in this signaling cascade, with LINC00511 playing a crucial regulatory role in this process. Knockdown of LINC00511 further enhances the inhibitory effect of β-elemene on this pathway, thereby contributing to the reversal of cisplatin resistance in A549/DDP cells.

### 3.6 LINC00511 regulates cisplatin resistance in A549/DDP cells via the PI3K/AKT/mTOR pathway: effects of PI3K inhibition

To further validate the role of LINC00511 in regulating the PI3K/AKT/mTOR signaling pathway, A549/DDP cells were treated with the PI3K inhibitor LY 294002. The cells were divided into nine groups: vector control, LINC00511 overexpression, LINC00511 knockdown, cisplatin + vector control, cisplatin + LINC00511 overexpression, cisplatin + LINC00511 knockdown, cisplatin + LY 294002, cisplatin + LY 294002 + LINC00511 overexpression, and cisplatin + LY 294002 + LINC00511 knockdown.

**FIGURE 6:**
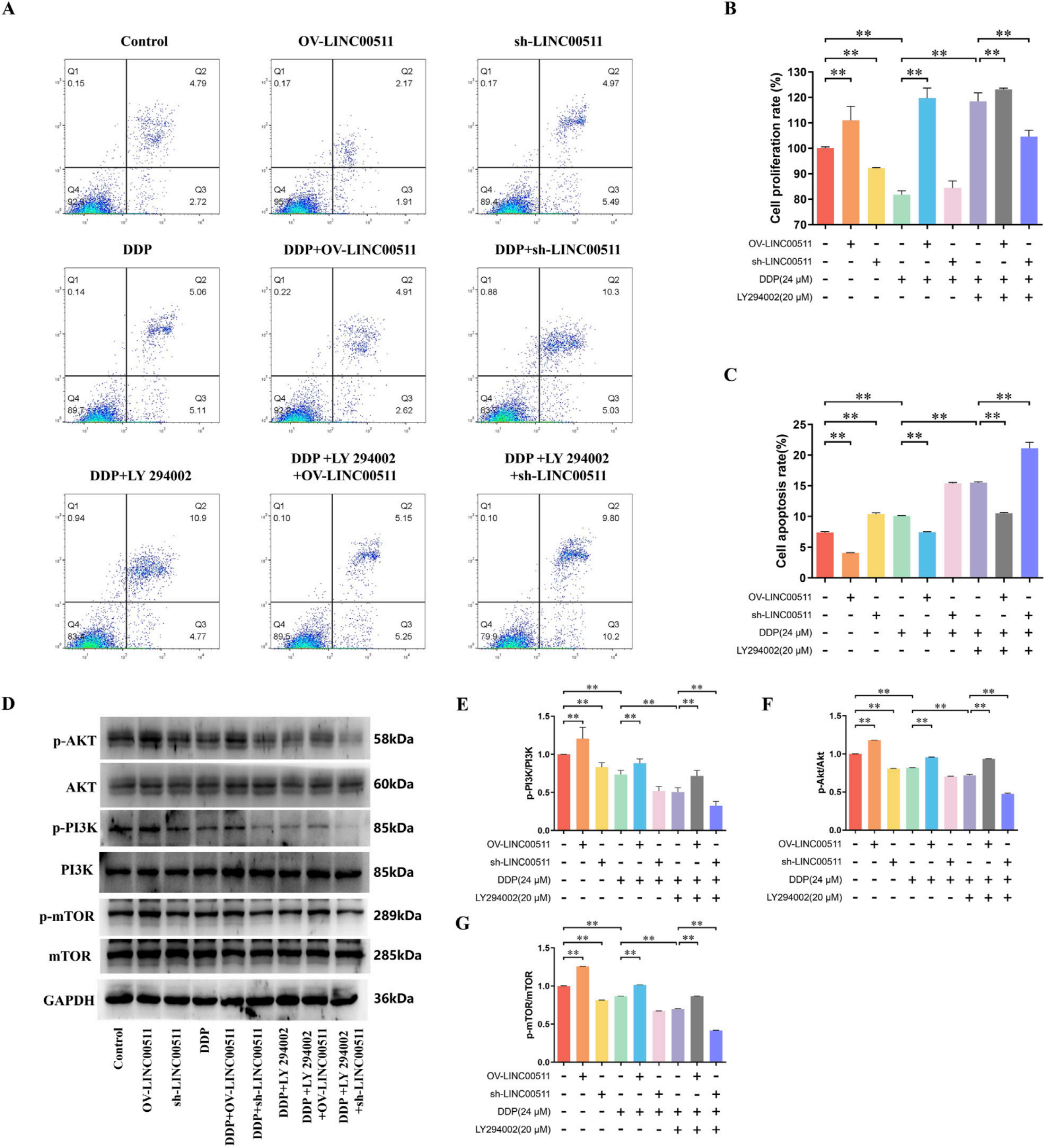
LINC00511 Regulates Cisplatin Resistance in A549/DDP Cells via the PI3K/AKT/mTOR Pathway. **(A,C)** Flow cytometry analysis of apoptosis in A549/DDP cells across different treatment groups; **(B)** CCK-8 assay showing the effects of LINC00511 on cell proliferation; **(D–G)** Western blot analysis of phosphorylated PI3K, AKT, and mTOR levels. Data are presented as mean ± SD from three independent experiments (biological replicates). ^**^ *P* < 0.01.

Flow cytometry analysis of apoptosis showed that LINC00511 overexpression significantly decreased apoptosis compared to the control, while LINC00511 knockdown resulted in a marked increase in apoptosis. Cisplatin treatment significantly increased apoptosis, and this effect was further amplified in the cisplatin + LINC00511 knockdown group. In contrast, apoptosis was reduced in the cisplatin + LINC00511 overexpression group compared to cisplatin alone. Notably, the combination of cisplatin and LY 294002 further enhanced apoptosis, with the most significant increase observed in the cisplatin + LY 294002 + LINC00511 knockdown group (*P* < 0.01) (Figures 6A,C). Cell proliferation, assessed by CCK-8, revealed that LINC00511 overexpression promoted cell proliferation, while LINC00511 knockdown reduced it. Cisplatin treatment alone led to a significant decrease in proliferation, which was further reduced in the cisplatin + LINC00511 knockdown group. However, LINC00511 overexpression mitigated the inhibitory effect of cisplatin on proliferation. LY 294002 treatment in combination with cisplatin resulted in a significant decrease in cell proliferation, and this effect was even more pronounced in the cisplatin + LY 294002 + LINC00511 knockdown group (*P* < 0.01) (Figure 6B). Western blot analysis showed no significant changes in the total levels of PI3K, AKT, and mTOR proteins across the groups. However, the levels of their phosphorylated forms (p-PI3K, p-AKT, p-mTOR) were significantly altered. LINC00511 overexpression increased the levels of phosphorylated PI3K, AKT, and mTOR, while LINC00511 knockdown decreased their phosphorylation. Cisplatin treatment reduced the levels of phosphorylated proteins, and this effect was further enhanced by the combination of cisplatin and LY 294002. The most pronounced reduction in phosphorylated PI3K, AKT, and mTOR levels was observed in the cisplatin + LY 294002 + LINC00511 knockdown group (*P* < 0.01), while the cisplatin + LY 294002 + LINC00511 overexpression group showed only a slight decrease in phosphorylation levels (Figures 6D–G).

These results demonstrate that LINC00511 modulates the PI3K/AKT/mTOR pathway in A549/DDP cells and that its knockdown enhances the pro-apoptotic and anti-proliferative effects of cisplatin and PI3K inhibition.

## 4 Discussion

The present study elucidates the molecular mechanism by which β-elemene enhances the sensitivity of lung adenocarcinoma cells to cisplatin, focusing particularly on the regulatory role of the long non-coding RNA LINC00511 and its downstream signaling effects. Our findings demonstrate that β-elemene suppresses cell proliferation and promotes apoptosis in cisplatin-resistant A549/DDP cells, with synergistic effects observed in combination with cisplatin. These findings reinforce the therapeutic potential of β-elemene in reversing cisplatin resistance and highlight LINC00511 as a pivotal molecular target.

Mechanistically, our data show that β-elemene significantly downregulates LINC00511 expression, leading to inhibition of the PI3K/AKT/mTOR signaling pathway—a well-established driver of chemoresistance in lung cancer (Glaviano et al., 2023). Knockdown of LINC00511 replicated these effects, and further sensitized cells to cisplatin-induced apoptosis, underscoring the causal relationship between LINC00511 expression and PI3K/AKT/mTOR activity. This was corroborated both *in vitro* and *in vivo*, where the combination of β-elemene and cisplatin yielded the most pronounced reduction in tumor burden and PI3K/AKT/mTOR phosphorylation. Notably, these molecular alterations did not affect the total protein levels of pathway components, suggesting that β-elemene exerts its effect primarily through modulating phosphorylation dynamics.

Our data add to the growing body of evidence implicating lncRNAs in modulating drug resistance. LINC00511, in particular, has emerged as an oncogenic lncRNA with roles in proliferation, metastasis, and therapy resistance in various cancers (Wang et al., 2020; Shi et al., 2021; Wang et al., 2022). By targeting this molecule, β-elemene disrupts a critical regulatory axis of survival signaling. These findings are especially relevant considering that other natural compounds, such as resveratrol (RVT), have demonstrated similar capabilities in overcoming resistance (Sheikh et al., 2024). For example, RVT has been shown to reverse tumor chemoresistance in lung cancer by inhibiting the PI3K/AKT/mTOR pathway, thereby enhancing cisplatin sensitivity—mechanistically consistent with our observations regarding β-elemene.

Moreover, it is important to contextualize our findings within the broader scope of natural compound research in lung cancer therapy. Isoflavones have been reported to inhibit NF-κB signaling, thereby reducing pro-inflammatory cytokine expression, a known contributor to tumor progression and resistance. In NSCLC, these compounds also regulate non-coding RNAs such as the circ 0031250/miR-873-5p/FOXM1 axis, thereby suppressing tumor cell migration and invasion (Ul Hassan et al., 2025). Although this axis was not directly investigated in our study, the similar mechanisms of action through non-coding RNA regulation underscore the therapeutic promise of phytochemicals like β-elemene.

It is noteworthy that while β-elemene and RVT both exert their effects via inhibition of the PI3K/AKT/mTOR pathway, their upstream regulatory targets may differ. The convergence of these compounds on a shared downstream signaling axis highlights a potential strategy for combinatorial or sequential therapeutic approaches that target distinct molecular nodes while converging on a common resistance pathway. Importantly, our findings suggest that interventions targeting both lncRNA expression and kinase signaling may be synergistic, providing a rationale for integrated therapeutic regimens.

Nevertheless, it is important to acknowledge a key limitation of this study. Although our findings suggest that LINC00511 may modulate the PI3K/AKT/mTOR signaling pathway, this conclusion is primarily drawn from correlative analyses using qRT-PCR and Western blot assays. These approaches reveal changes in gene and protein expression but do not establish direct molecular interactions. Mechanistic validation through assays such as RNA immunoprecipitation (RIP), chromatin isolation by RNA purification (ChiRP-Seq), luciferase reporter assays, or RNA pull-down experiments were not performed in this study. Therefore, the regulatory relationship between LINC00511 and the PI3K/AKT/mTOR pathway remains correlative. Our study establishes a consistent association, but future work is needed to confirm a direct causal interaction. Future studies employing these mechanistic assays will be essential to confirm whether LINC00511 directly interacts with key components of the PI3K/AKT/mTOR signaling cascade or modulates its activity via intermediates. Clarifying this interaction will provide deeper insight into the molecular underpinnings of cisplatin resistance and strengthen the rationale for targeting LINC00511 in therapeutic strategies.

## 5 Conclusion

In conclusion, our study demonstrates that β-elemene effectively reverses cisplatin resistance in lung adenocarcinoma by downregulating the long non-coding RNA LINC00511, thereby suppressing the PI3K/AKT/mTOR signaling pathway. Our results indicate that β-elemene significantly enhances cisplatin-induced apoptosis and inhibits proliferation in both cisplatin-sensitive A549 and resistant A549/DDP cells, with synergistic effects observed in combination therapy. Mechanistically, β-elemene reduces LINC00511 expression, and knockdown of LINC00511 further augments the pro-apoptotic and anti-proliferative responses to cisplatin and β-elemene, while its overexpression counteracts these effects. *In vivo* xenograft models confirm that the combination treatment markedly reduces tumor growth, decreases Ki-67 proliferation markers, increases apoptosis, and downregulates LINC00511 expression. Additionally, β-elemene and LINC00511 modulation specifically affect the phosphorylation levels of PI3K, AKT, and mTOR without altering total protein expression, highlighting the pathway’s role in mediating resistance. The use of the PI3K inhibitor LY294002 further validates that LINC00511 regulates cisplatin resistance through this pathway. Collectively, our results identify β-elemene as a promising therapeutic agent for overcoming cisplatin resistance in lung cancer by targeting the LINC00511/PI3K/AKT/mTOR axis.

## Data Availability

Original datasets are available in a publicly accessible repository: The original contributions presented in the study are publicly available. This data can be found here: https://cstr.cn/31253.11.sciencedb.32458, and https://cstr.cn/31253.11.sciencedb.32336.
